# Supplementary material for: Marine Bacterium Vibrio sp. CB1-14 Produces Guanidine Alkaloid 6-epi-Monanchorin, Previously Isolated from Marine Polychaete and Sponges
Source: Mar Drugs. 2019 Apr 4;17(4):213. doi: 10.3390/md17040213 (PMC6521263; doi:10.3390/md17040213)
Supplement: Supplementary file 1 [file marinedrugs-17-00213-s001.pdf]

S1 Supplementary data contents page

Title: Marine bacterium *Vibrio* sp. CB1-14 Produces Guanidine Alkaloid 6-*epi*-Monanchorin, Previously Isolated from Marine Polychaete and Sponges.

Authors: Tatyana Makarieva*, Larisa Shubina, Valeria Kurilenko, Marina Isaeva, Nadezhda Chernysheva, Roman Popov, Evgeniya Bystritskaya, Pavel Dmitrenok, and Valentin Stonik

Address: G.B. Elyakov Pacific Institute of Bioorganic Chemistry (PIBOC), Russian Academy of Sciences, Prospect 100 let Vladivostoku, 159, Vladivostok 690022, Russia; makarieva@piboc.dvo.ru (T.M.); shubina@piboc.dvo.ru (L.S.); valerie@piboc.dvo.ru (V.K.); issaeva@piboc.dvo.ru (M.I.); chernysheva.nadezhda@gmail.com (N.C.); prs_90@mail.ru, (R.P.); belyjane@gmail.com (E.B.); paveldmt@piboc.dvo.ru; (P.D.); stonik@piboc.dvo.ru (V.S.)

Contents:

S2 HRESIMS spectra of 6-epi-monanchorin (**2**) isolated from polychaete *Chaetopterus variopedatus* and marine bacterium Vibrio sp. CB1-14;

S3 ^{1}H NMR spectra for 6-epi-monanchorin (**2**) isolated from polychaete *Chaetopterus variopedatus* and marine bacterium *Vibrio* sp. CB1-14;

S4 HPLC chromatograms of culture medium extract and cells extracts of CB1-14.

S5 Table S1. Accession numbers and Chimera identification of 16S rRNA of bacterial isolates

S2 HRESIMS spectrum of 6-epi-monanchorin (**2**) isolated from marine polychaete *Chaetopterus variopedatus*

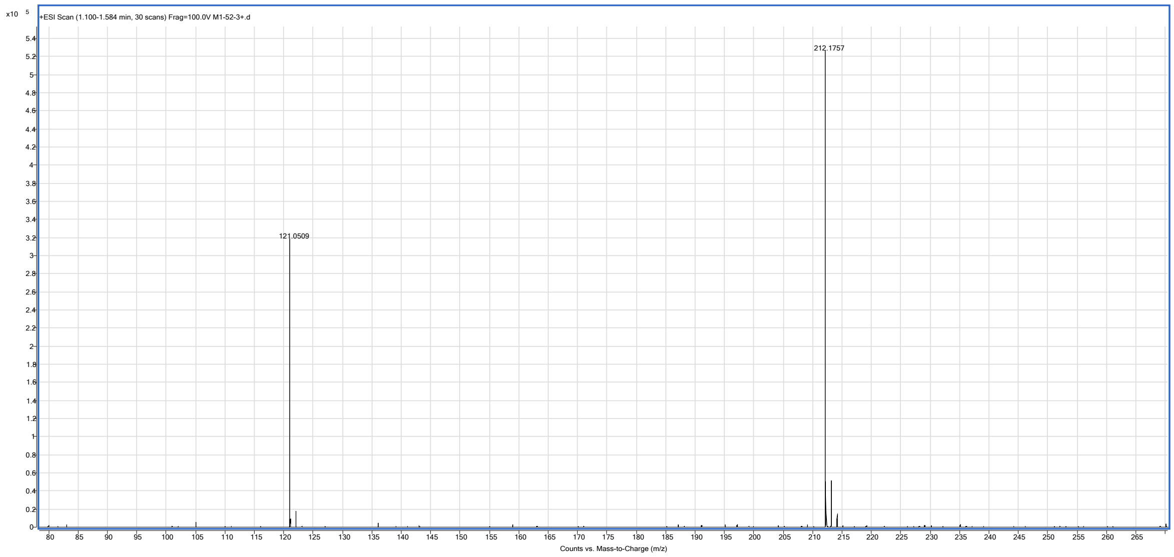

HRESIMS spectrum of 6-epi-monanchorin (**2**) isolated from marine bacterium *Vibrio* sp. CB1-14

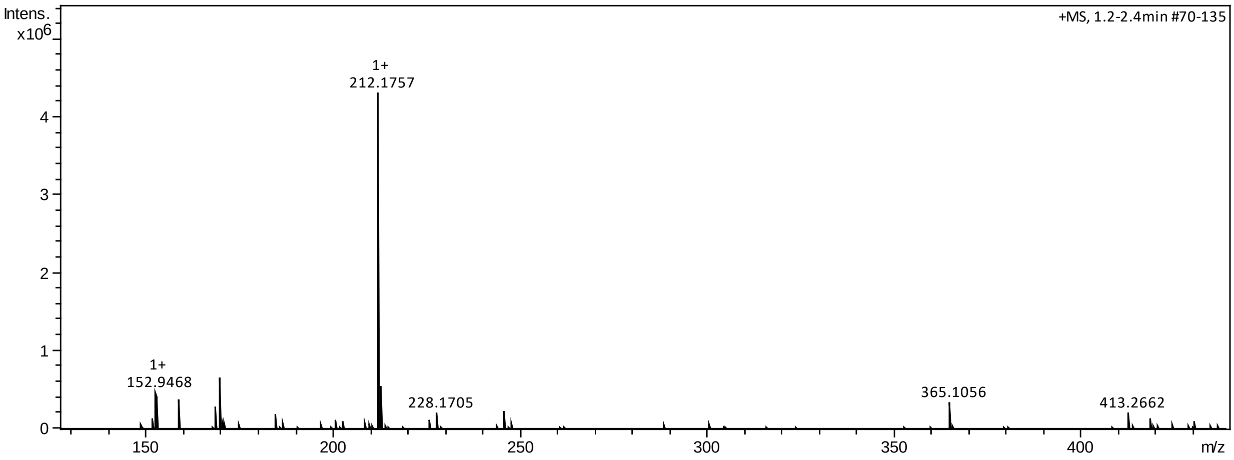

S3

Comparison of ^{1}H NMR spectra for 6-epi-monanchorin (**2**) isolated from polychaete *Chaetopterus variopedatus* (**red**) and marine bacterium *Vibrio* sp. CB1-14 (**blue**)

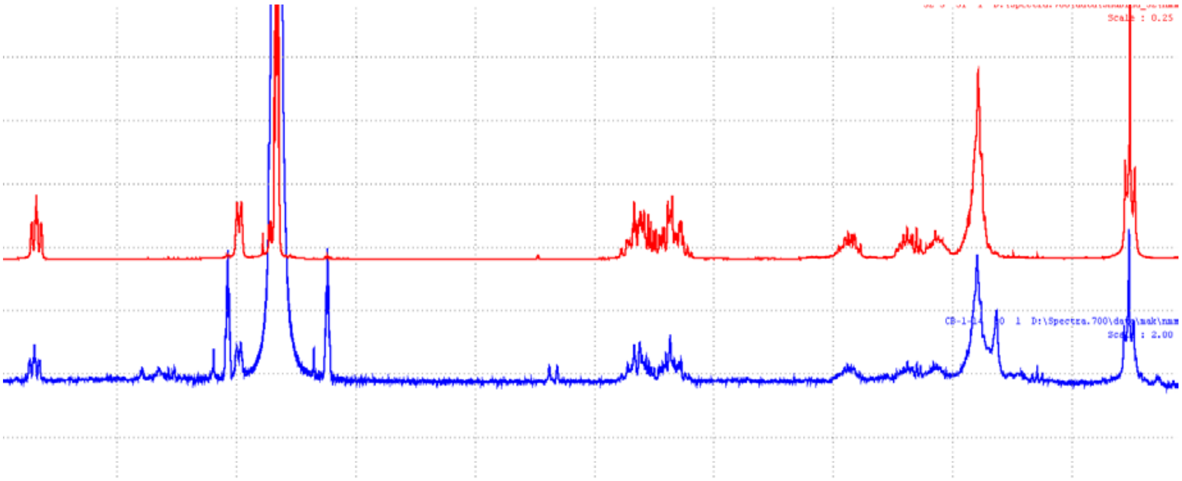

S4 HPLC chromatogram of culture medium extract of CB1-14.

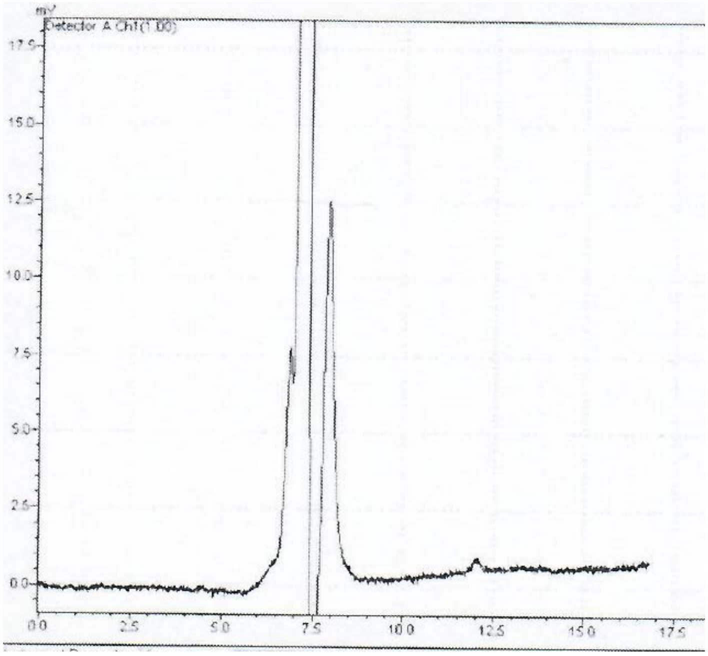

HPLC chromatogram of cells extract of CB1-14.

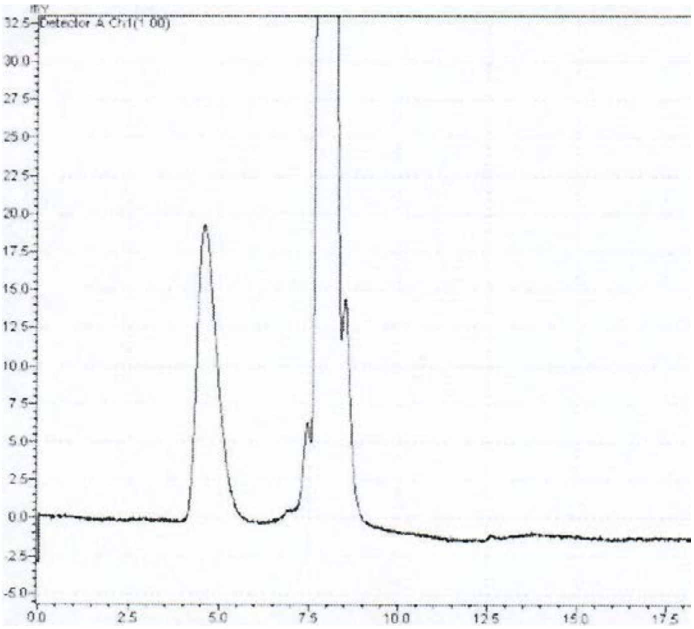

S5

Table S1. Accession numbers and Chimera identification of 16S rRNA of bacterial isolates

Name of isolates	GenBank accession no	Chimera identification*	
		Group	Result
CB2-12	MK598712	Vibrio	Not deciphered to be a chimera
CB2-5	MK598713	Vibrio	Not deciphered to be a chimera
CB2-8	MK598714	Vibrio	Not deciphered to be a chimera
CB2-10	MK598715	Vibrio	Not deciphered to be a chimera
CB1-5	MK598716	Vibrio	Not deciphered to be a chimera
CB2-1	MK598717	Vibrio	Not deciphered to be a chimera
CB1-11	MK598718	Vibrio_shilonii	Not deciphered to be a chimera
CB2-9	MK598719	Vibrio_shilonii	Not deciphered to be a chimera
CB2-11	MK598720	Vibrio_shilonii	Not deciphered to be a chimera
CB1-6	MK598721	Vibrio	Not deciphered to be a chimera
CB2-7	MK598722	Vibrio	Not deciphered to be a chimera
CB1-13	MK598723	Bacillus_hwajinpoensis	Not deciphered to be a chimera
CB1-18	MK598724	Bacillus_hwajinpoensis	Not deciphered to be a chimera
CB1-3	MK598725	Stappia_f	Not deciphered to be a chimera
CB1-1	MK598726	Vibrio_shilonii	Not deciphered to be a chimera
CB2-13	MK598727	Vibrio_shilonii	Not deciphered to be a chimera
CB2-4	MK598728	Vibrio_shilonii	Not deciphered to be a chimera
CB1-10	MK598729	Vibrio_shilonii	Not deciphered to be a chimera
CB1-7	MK598730	Vibrio	Not deciphered to be a chimera
CB1-14	MK598731	Vibrio	Not deciphered to be a chimera

* ES Wright *et al.* (2012) "DECIPHER, A Search-Based Approach to Chimera Identification for 16S rRNA Sequences." *Applied and Environmental Microbiology*, doi:10.1128/AEM.06516-11.
